# AI-Powered Early Detection of Sepsis in Emergency Medicine

**DOI:** 10.3390/life15101576

**Published:** 2025-10-10

**Authors:** Sergey Aityan, Rolando Herrero, Abdolreza Mosaddegh, Haitham Tayyar, Ebunoluwa Adebesin, Sai Pranavi Jeedigunta, Hangyeol Kim, Manuel Mersini, Rita Lazzaro, Nicola Iacovazzo, Ciro Gargiulo Isacco

**Affiliations:** 1 Department of Multidisciplinary Engineering, Northeastern University, Oakland, CA 94613, USA; s.aityan@northeastern.edu (S.A.); a.mosaddegh@northeastern.edu (A.M.); adebesin.e@northeastern.edu (E.A.); kim.hang@northeastern.edu (H.K.); 2 Department of Multidisciplinary Engineering, Northeastern University, Boston, MA 02115, USA; h.tayyar@northeastern.edu (H.T.); jeedigunta.s@northeastern.edu (S.P.J.); 3 Biovitalage S.R.L., 70010 Valenzano, Italy; manuelmersini@gmail.com; 4 Territorial Emergency System, SET 118, 74121 Taranto, Italy; rita-lazzaro@libero.it; 5 Territorial Center for Medical Assistance, 74121 Taranto, Italy; iacovazzonicola@gmail.com; 6 Department of Interdisciplinary Medicine (DIM), Aldo Moro University of Bari, 70121 Bari, Italy; drciroisacco@gmail.com

**Keywords:** sepsis, machine learning, white-box ML, black-box ML, urgent care, ambulance-based detection, hospital setting

## Abstract

Sepsis remains a critical medical emergency caused by a dysregulated immune response to infection, with timely detection and intervention being essential for improving survival rates. Traditional methods often rely on clinician intuition and structured scoring systems, which may be time-intensive and prone to variability. To address these limitations, *Machine Learning* (ML) offers a powerful alternative, bringing precision and efficiency to sepsis detection. This study investigates both white-box and complex black-box ML models applied to patient data collected across the continuum of care, including monitoring at the urgent care, en route in ambulances, and diagnostics conducted within hospital emergency department settings themselves. White-box models, such as logistic regression and decision trees, are valued for their interpretability, allowing healthcare providers to understand and trust the reasoning behind predictions. Meanwhile, black-box models like deep neural networks and support vector machines deliver superior accuracy but pose challenges in clinical transparency. This trade-off between explainability and performance is explored in detail, supported by experimental results aimed at identifying the most effective computational strategies for early sepsis recognition across diverse healthcare environments.

## 1. Introduction

Recent developments in AI models have catalyzed significant advancements in emergency medicine, particularly in the early detection of life-threatening conditions such as sepsis [1,2]. Sepsis is a critical, systemic response to infection that, if left untreated, can escalate into septic shock and result in death. It can originate from several sources, including

- Lungs;
- Kidneys, bladder, and other parts of the urinary system;
- Digestive system;
- Bloodstream;
- Wounds or burns;
- COVID-19.

In this study, a single dataset containing 140 entries (evenly divided between patients diagnosed with sepsis and those without) is used. These patient data comes from cases in the territorial medicine area between University of Rome Tor Vergata and Policlinico di Bari in Puglia, Italy. For each individual, sepsis detection is recorded across three key care settings: *at urgent care*, *in the ambulance*, and *in the hospital emergency department*. Specifically, in the context of this paper, patients in need of urgent or emergency medical attention may seek care from a regular physician, visit an urgent care center, call for an ambulance, or go directly to a hospital emergency department, each offering varying depths of diagnostic testing and clinical evaluation. In some cases, a physician may initiate ambulance transport to expedite hospital care. To ensure comprehensive coverage across the spectrum of early intervention, our research evaluated all three tiers of medical services, urgent care, ambulance, and hospital emergency departments, for their roles in the early prediction of sepsis. Note that when compared to other datasets, ours is unique in that it integrates multiple sequential stages of patient care, capturing the full continuum typical of emergency medicine workflows.

The dataset includes a rich combination of epidemiological and clinical features, such as demographics, vital signs, symptoms (i.e., fever, confusion, hypotension), and laboratory results (i.e., creatinine, lactate). By applying both white-box and black-box AI techniques, the analysis aims to identify features most predictive of sepsis onset and to understand how the detection context affects diagnostic accuracy.

This integrated, real-world dataset offers unique insights into the progression and management of sepsis across multiple stages of emergency care, highlighting the potential of AI to enhance early intervention strategies.

Although our dataset is balanced for modeling purposes, real-world sepsis prevalence is significantly lower, which raises important considerations for clinical applicability. To contextualize our approach, we note that prior studies have focused on targeted biomarkers such as the LIP score and molecular mechanisms like noncoding RNAs in sepsis-associated organ injury, whereas our work introduces a multistage framework that reflects the complexity of emergency care and supports broader diagnostic generalization.

The remainder of the paper is organized as follows: related work is introduced in Section 2, an overview and an analysis of the datasets are respectively presented in Section 3 and Section 4, and conclusions and future work are provided in Section 5.

## 2. Literature Review, Motivation and Contribution

AI-driven sepsis prediction has gained substantial traction in recent years, with model performance largely dependent on dataset quality, diversity, and contextual richness. A wide spectrum of ML techniques, from traditional algorithms to advanced *Deep Learning* (DL) architectures, has demonstrated strong predictive power across various clinical scenarios.

Studies have employed models such as *Logistic Regression* (LR), *Random Forest* (RF), *Support Vector Machine* (SVM), and *Extreme Gradient Boosting* (XGBoost). For instance, RF achieved 77.5% accuracy in [3], and 99.01% with an *Area Under the Curve* (AUC) of 99.99% in [4], effectively forecasting sepsis 24 h before clinical diagnosis. Gradient boosting techniques in [5] attained an AUC of 0.91 and F1-score of 87%, while balanced bagging and SVM models reached up to 98% accuracy in [6].

The use of time-series data and enhanced preprocessing has proven beneficial, as shown in [7], where *artificial neural networks* (ANNs), *Convolutional Neural Networks* (CNNs), and *Long Short-Term Memory* (LSTM) networks improved early detection. XGBoost achieved 95.01% accuracy in [8]. Nontraditional modalities such as thermal imaging with SVM [9], blockchain-integrated ANNs [10], and semi-supervised learning via SPSSOT [11] contributed to mortality prediction and hospital-wide detection, respectively.

Deep learning models in [12,13] leveraged LSTM and immune biomarkers to reach 96.26% accuracy and 99.54% AUC. Synthetic data generation and augmentation enhanced neonatal sepsis prediction in [14], where XGBoost achieved an AUROC of 0.91, with transfer learning yielding marginal LSTM improvements.

In hospital contexts, studies like [15,16] used gradient-boosted trees and RF to predict sepsis up to 15 h before onset, achieving AUROCs of 0.743–0.777 during testing and accuracies above 99% with ensemble methods. Architecture choices in [17] influenced performance ranges between 0.6 and 0.9. CNNs in [18] delivered superior feature extraction from sparse datasets, attaining 99.99% accuracy with full feature sets.

Mortality prediction has been explored using MLP models [19], which achieved AUROC of 0.835 using demographic and *Sequential Organ Failure Assessment* (SOFA) score features. In [20], preprocessing techniques like imputation and transformation helped RF reach 96% accuracy using the PhysioNet Sepsis Challenge dataset.

Temporal feature modeling was central to [21], where a multi-subset XGBoost framework captured trends via delta values and statistical metrics, yielding AUROC scores of 0.7906 for early prediction and 0.9199 for septic shock. Pediatric diagnostics in [22] leveraged gene expression via LIMMA on GSE66099, with RF attaining 91.79% accuracy and 100% specificity. HHO in [23] further improved gene-based pediatric classification, enabling SVM to achieve 96.64% accuracy and 0.9575 AUROC.

Other studies tackled class imbalance with techniques such as SMOTE [24], where XGBoost yielded 0.95 accuracy and 0.978 AUROC in peripheral healthcare settings. Interpretability was emphasized in [25] via SHAP and XGBoost, identifying features like GCS, ventilation status, and SAPS II as key mortality indicators. Feature selection via GLM [26] and importance ranking through XGBoost [27] improved ANN and MLP models across both PhysioNet and MIMIC-III datasets.

Table 1 presents a comparative overview of ML models applied to early sepsis detection, consolidating performance metrics such as accuracy, AUC, precision, and recall from multiple recent studies. The table highlights the strong predictive capabilities of ensemble-based methods like random forest and XGBoost, especially when combined with sampling techniques like SMOTE. Deep learning models such as CNNs and LSTMs exhibit high accuracy, particularly when applied to rich clinical datasets, while optimization and feature selection approaches, such as Harris hawks optimization and SHAP, contribute to enhanced interpretability and generalization in pediatric and ICU-specific scenarios. This summarized benchmark supports informed selection of models for both high-performance prediction and context-specific deployments.

Contributions of This Work: Unlike previous studies relying on a single clinical setting or modality, the research presented in this paper integrates patient data from three critical stages, *at urgent care*, *in the ambulance*, and *in the hospital emergency department*. This multi-stage, real-world perspective offers a robust foundation for early sepsis detection through both interpretable white-box models and high-performing black-box models. By evaluating prediction across care transitions and leveraging complementary datasets, this study provides a comprehensive approach to modeling sepsis in emergency medical systems.

## 3. The Datasets

The dataset employed in this study comprises 140 patient records, evenly divided between sepsis-positive and sepsis-negative cases. What distinguishes this dataset is its structured focus on the continuum of care, capturing key data points across three critical stages of patient management: *at urgent care*, *in the ambulance*, and *in the hospital emergency department*. By weaving these layers into a coherent temporal sequence, the dataset mirrors real-world emergency workflows and enables us to trace the evolution of sepsis indicators, from subtle symptoms in the urgent care environment to definitive hospital emergency department diagnostics. This structure provides an ideal proving ground for AI models, facilitating the evaluation of their effectiveness in detecting and predicting sepsis at each critical juncture in patient care. The following subsections delve into the specifics of each stage.

### 3.1. Urgent Care

The *urgent care assessment dataset* represents the initial phase in the patient monitoring continuum and captures symptom data prior to any formal medical or ambulance intervention. At this stage, objective clinical measurements are generally unavailable; however, structured symptom scoring provides early indicators of systemic deterioration, particularly in relation to sepsis. The dataset includes a binary sepsis outcome (YES/NO) and subjective assessments of symptom severity using a standardized scale ranging from 0 (normal or absent) to 3 (severe or life-threatening). This early information plays a critical role in identifying at-risk individuals before hospital emergency department contact.

The severity of symptoms is numerically scored (Table 2). For example, fever is classified as follows: a score of 0 indicates no fever (temperature below 37.5 °C), 1 corresponds to mild fever (37.5–38.0 °C), 2 signifies moderate fever (38.1–39.0 °C), and 3 reflects high fever (above 39.0 °C). Similarly, breathing difficulty is scored as 0 for normal breathing, 1 for mild difficulty during exertion, 2 for shortness of breath at rest, and 3 for severe respiratory distress requiring support. This consistent scoring approach across symptoms enables the standardized evaluation of patient status in pre-hospital settings.

A typical example from the dataset includes a patient with a fever severity of 2, altered mental status severity of 1, palpitations severity of 0, chills severity of 2, breathing difficulty severity of 1, skin discoloration severity of 0, fatigue severity of 2, and confusion severity of 0, with a sepsis diagnosis marked as YES. In this scenario, the presence of moderate fever, chills, and fatigue, alongside mild breathing difficulty, suggests an early inflammatory response, possibly indicating the onset of sepsis.

Clinically, the urgent care assessment dataset supports decision-making for early triage by quantifying subjective indicators of illness before any physiological data are available (see Table 3). Its structured design enables integration into remote monitoring systems and telehealth platforms, paving the way for seamless transition across stages of care, from urgent care to ambulance to hospital. By improving data continuity and reducing the time to intervention, this dataset helps clinicians and automated models identify patients at risk of deterioration in non-clinical settings.

### 3.2. Ambulance

The *Ambulance Assessment Dataset* represents the intermediate stage in patient monitoring, capturing clinical and observational features recorded during patient transport to the hospital emergency department. Observational features documented in this stage are identical to those assessed at urgent care but are re-evaluated during ambulance transit, as patient symptoms may have worsened since the initial assessment. The inclusion of clinical measurements during this phase enriches the dataset with physiological data that enhance the precision of symptom interpretation and provide a clearer understanding of clinical deterioration. This combination of subjective severity scores and objective clinical indicators allows for early identification of sepsis and contributes significantly to predictive modeling.

In this stage, symptom severity is assessed using a structured scale from 0 to 3. For example, fever scoring ranges from no fever (temperature below 37.5 °C, score 0) to high fever (above 39.0 °C, score 3). Similarly, breathing difficulty is classified from normal respiration (score 0) to severe respiratory distress requiring support (score 3). This standardized scoring enables comparability across different patients and facilitates consistent monitoring of symptom evolution during transit (see Table 4).

To illustrate, consider the following sample patient record obtained during ambulance transport: fever severity of 3, altered mental status score of 1, palpitations score of 1, chills score of 1, breathing difficulty score of 1, and skin abnormality score of 1. Vital signs recorded include systolic blood pressure of 83 mmHg, diastolic pressure of 59 mmHg, heart rate of 131 bpm, and respiratory rate of 33.5 breaths per minute. The patient’s oxygen saturation is 94.26%, glucose level is 138.87 mg/dL, capillary refill time is 3.35 s, and the Glasgow Coma Scale score is 14.51. The recorded temperature is 38.90°C, and the sepsis diagnosis is marked as YES.

The example in Table 5 demonstrates a constellation of abnormal vital signs and symptom scores indicative of progressive systemic dysfunction. The patient’s low blood pressure, elevated heart and respiratory rates, and prolonged capillary refill time signal cardiovascular and respiratory compromise. Combined with a high fever and altered mental status, these features strengthen the case for early sepsis detection.

Clinically, the ambulance dataset offers critical insights during a pivotal transition period. It captures dynamic changes in patient condition and augments symptom-based data with objective metrics necessary for accurate triage. The availability of this information prior to hospital arrival enables rapid clinical decision-making and the deployment of predictive models that improve time-to-intervention and reduce sepsis-associated morbidity and mortality.

### 3.3. Hospital Emergency Department

The *hospital emergency department assessment dataset* constitutes the final and most critical stage of patient monitoring within the sepsis evaluation continuum. It encompasses a rich combination of subjective symptom severity scores, objective clinical assessments, and advanced laboratory biomarkers collected during inpatient care. At this stage, patients typically present with their most severe symptoms and are undergoing comprehensive diagnostic and therapeutic intervention. The dataset is designed to capture both clinical deterioration and systemic dysfunction with high granularity, enabling the identification and management of severe sepsis cases.

Symptom severity within the dataset is scored on a standardized 0 to 3 scale, where 0 represents normal status and 3 denotes a severe or life-threatening presentation (see Table 6). This applies to symptoms such as fever, breathing difficulty, fatigue, confusion, and altered mental status. Clinical assessments, including the SOFA score and auscultation, are similarly rated, enabling quantification of organ dysfunction and respiratory abnormalities. For example, fever severity ranges from no fever below 37.5 °C to high-grade fever exceeding 39.0 °C, while the SOFA score ranges from no organ dysfunction to severe failure associated with elevated mortality risk. These scoring systems support uniform interpretation across diverse patient records.

The dataset also incorporates high-resolution laboratory markers and blood gas metrics that provide insight into inflammatory status, tissue oxygenation, and metabolic balance. White blood cell count, lactate level, creatinine, bilirubin, and procalcitonin are essential for diagnosing systemic infection and organ compromise. Arterial blood gas parameters, including pH, pCO_2_, and pO_2_, further inform respiratory and acid-base status. Microbiological confirmation through blood culture adds a diagnostic endpoint to guide therapy.

Table 7 shows an example hospital emergency department record from the dataset includes a fever severity of 3, altered mental status score of 2, palpitations and chills both rated at 2, breathing difficulty scored at 2, fatigue at 3, and confusion at 2. The auscultation score is 2 and the detailed medical history score is 3. Laboratory values reveal white blood cell count of 20.026 × 10^3^/µL, lactate level of 4.024 mmol/L, C-reactive protein of 115.001 mg/L, procalcitonin of 3.437 ng/mL, creatinine of 2.147 mg/dL, bilirubin of 2.081 mg/dL, and platelet count of 121.39 × 10^3^/µL. The SOFA score is recorded as 4.802. Arterial blood gas results show a pH of 7.254, pCO_2_ of 42.161 mmHg, and pO_2_ of 55.201 mmHg. Blood cultures return negative, and the sepsis diagnosis is confirmed (SEPSIS = Yes).

This patient profile illustrates the severity of sepsis as captured during hospital emergency department evaluation. Elevated inflammatory markers, metabolic acidosis, and evidence of organ dysfunction reflect advanced disease progression. The comprehensiveness of this dataset enables AI models to learn the clinical arc of sepsis development, from early warning signs to terminal complications, thereby improving predictive capabilities and optimizing care pathways.

## 4. Analysis of Datasets

This section integrates data collected across the full continuum of patient care, at urgent care, in the ambulance, and in the hospital emergency department, to predict the presence and progression of sepsis at each critical stage. The predictive framework relies on a modeling approach that includes white-box models and more complex black-box models.

### 4.1. White Box Analysis

White-box ML models, including logistic regression, decision trees, and random forests, enable interpretable and clinically meaningful predictions when applied across the structured datasets collected in the urgent care, ambulance, and hospital settings. Logistic regression offers transparent coefficients that elucidate the influence of features such as heart rate, oxygen saturation, and timing of care across all three stages, helping quantify their contribution to outcomes like hospital admission or mortality. Decision trees emulate clinical logic by constructing traceable decision paths that reflect diagnostic reasoning from initial urgent care symptom monitoring through ambulance triage to hospital emergency department evaluations. While random forests introduce greater complexity through ensemble learning, they preserve interpretability via feature importance scores, highlighting variables that consistently signal risk or recovery across the continuum of care.

#### 4.1.1. Urgent Care

White-box ML models, logistic regression, decision tree, and random forest, were applied to the urgent care assessment dataset to evaluate their effectiveness in predicting sepsis based on structured symptom severity scores. All input features were standardized using *StandardScaler*, and performance was evaluated using an 80/20 stratified train-test split combined with five-fold stratified cross-validation.

**Figure 1:**
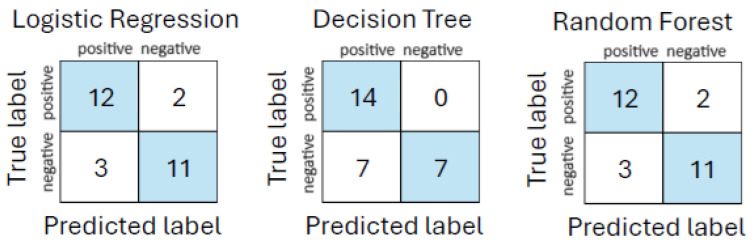
Confusion matrices for models on urgent care assessment dataset.

The initial performance evaluation revealed that both logistic regression and random forest classifiers achieved accuracies of 82% and AUC values of 90%, outperforming decision tree, which yielded 75% accuracy and 83% AUC. These results are summarized in Table 8. Similarly, Figure 1 shows the corresponding confusion matrices.

Cross-validation yielded slightly lower metrics. Logistic regression produced a mean accuracy of 0.69 and mean AUC of 0.73. Decision tree had higher consistency, with a mean accuracy of 0.78 and AUC of 0.87, while random forest reached 0.74 accuracy and 0.86 AUC.

**Figure 2:**
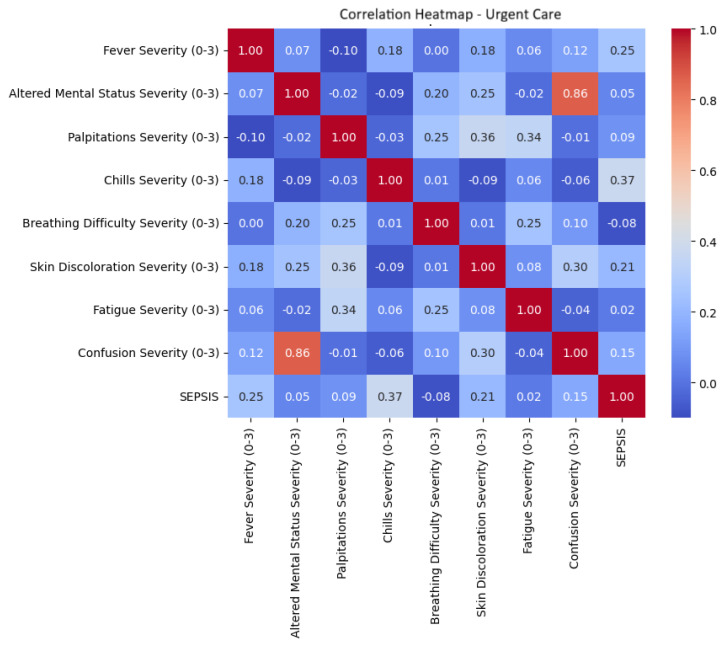
Correlation heatmap—urgent care assessment.

To further understand the relationship between each feature and the target variable, correlation coefficients were computed between symptom severity scores and the binary sepsis diagnosis. As visualized in Figure 2, fever severity exhibited the strongest correlation (0.37), followed by chills severity (0.25) and skin discoloration severity (0.21).

Feature importance rankings from each model reinforce these findings. Fever severity was consistently ranked as the top predictor in both decision tree and random forest models. Logistic regression, while more sensitive to linear effects, highlighted chills severity with the highest positive coefficient. The top features across the three models are presented in Table 9.

Interpretation of the results indicates that decision tree and random forest models were more effective than logistic regression in capturing nonlinear relationships among features, as reflected by their superior AUC scores. The decision tree model demonstrated balanced precision and recall, while random forest offered robust generalization. Logistic regression, while easier to interpret, struggled to model complex interactions in the symptom space.

Clinically, these findings affirm the utility of structured, interpretable models in the early detection of sepsis in urgent care settings. Features such as fever, chills, and breathing difficulty consistently emerged as high-value predictors across all models. Their reproducibility enhances trust and transparency, aligning well with clinical workflows that demand both accuracy and explainability.

#### 4.1.2. Urgent Care and Ambulance Analysis

White-box classification models were applied to a combined dataset integrating symptom severity scores from the urgent care environment and vital signs, clinical observations, and repeated symptoms collected during ambulance transport. This enriched dataset provided a deeper and more representative view of each patient’s condition during early medical assessment. All features were standardized, and model evaluation was performed using stratified cross-validation to ensure robustness across balanced sepsis-positive and sepsis-negative cases.

**Figure 3:**
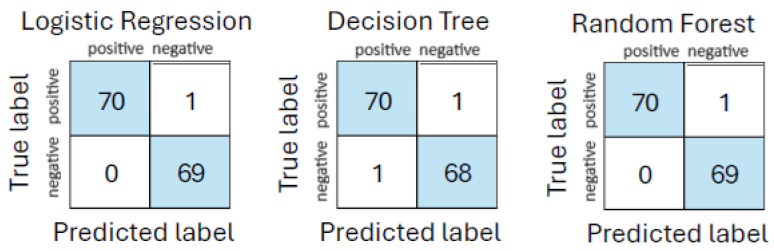
Confusion matrices for models on urgent care + ambulance dataset.

The performance comparison showed high predictive accuracy across all three models. logistic regression and random forest both achieved a mean accuracy of 99%, with logistic regression attaining an AUC of 98% and random forest slightly higher at 99%. The Decision Tree classifier followed closely, with an accuracy of 98% and AUC of 98%. These results, summarized in Table 10, confirm the excellent performance of white-box models in sepsis detection when richer physiological context is added. The corresponding confusion matrices are shown in Figure 3.

**Figure 4:**
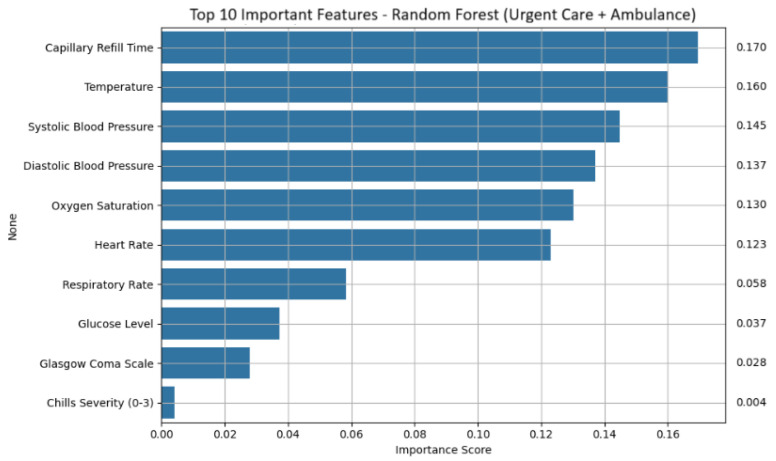
Top 10 important features: random forest (urgent care + ambulance).

Feature importance analysis revealed a consistent set of physiological and symptom-based indicators that contributed most strongly to sepsis prediction (shown in Figure 4 for random forest). Across models, capillary refill Time emerged as the dominant feature, reflecting impaired perfusion often observed in septic patients. Temperature was also consistently influential, suggesting that fever remains a reliable signal even in early mobile assessments. Blood pressure metrics, both systolic and diastolic, were important, especially for random forest and logistic regression, with higher pressure values negatively correlated with sepsis likelihood. Additional key features included oxygen saturation, heart rate, respiratory rate, glucose level, and Glasgow coma scale. Symptom severity scores, such as chills severity, were also retained as meaningful predictors.

Table 11 presents the ranked importance of key features based on random forest output, while Table 12 summarizes the influence direction and magnitude derived from the logistic regression model.

Notably, the decision tree model prioritized temperature as the primary decision node, with minimal use of other features, indicating a high reliance on fever as a threshold indicator. Glucose level showed limited impact, suggesting that metabolic disturbance may vary in clinical relevance at this stage.

Collectively, the white-box models demonstrated that integrating urgent care and ambulance-stage data allows for highly accurate and interpretable prediction of sepsis. The clinical utility of features such as capillary refill time, temperature, and blood pressure reinforces their value in early triage workflows and supports their inclusion in remote screening tools or paramedic protocols. The combination of high sensitivity and model transparency makes these models suitable for integration into real-time decision support systems across emergency care transitions.

#### 4.1.3. Urgent Care, Ambulance and Hospital Emergency Department Analysis

**Figure 5:**
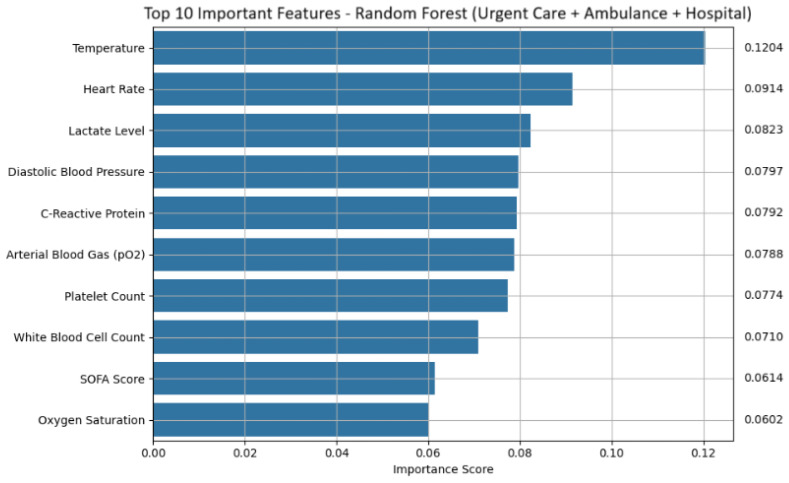
Top 10 important features: random forest (urgent care + ambulance + hospital emergency department).

To assess the performance of interpretable ML models across the full care continuum, white-box classifiers were trained and evaluated using data aggregated from all three stages of emergency assessment: urgent care, ambulance, and hospital. This integrated dataset includes symptom severity scores, physiological measurements, clinical observations, laboratory biomarkers, and organ function indicators. Each model was assessed using cross-validation, with results summarized in Table 13. Feature standardization was applied using *StandardScaler* to ensure consistent scaling across diverse input sources. The matching confusion matrices are shown in Figure 5.

Logistic regression performed with exceptional accuracy, misclassifying only one out of 140 samples while maintaining high interpretability via feature coefficients. The decision tree model was slightly less accurate, with two misclassifications, but remains valuable due to its intuitive rule-based structure. Random forest achieved the highest AUC (99.23%), demonstrating strong generalization and offering robust feature importance outputs.

**Figure 6:**
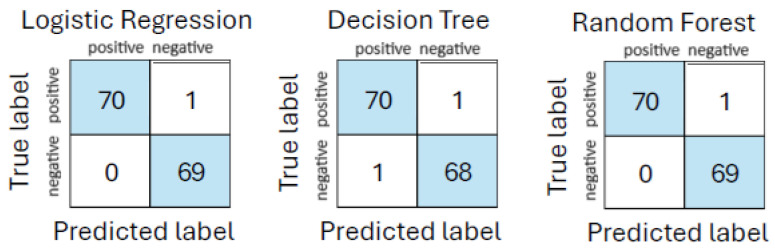
Confusion matrices for models on urgent care + ambulance + hospital emergency department dataset.

Feature attribution analysis revealed key predictors across models. Random forest placed highest importance on Temperature, Heart Rate, Lactate Level, and Diastolic Blood Pressure, features that reflect metabolic stress and hemodynamic compromise (shown in Figure 6 and Table 14 for random forest). Additional predictors such as C-reactive protein, white blood cell count, platelet count, SOFA score, and arterial blood gas pO_2_ values contributed to robust prediction. These features are biologically meaningful and align with established clinical indicators of sepsis.

Logistic regression coefficients further clarified directional influences. Positive coefficients indicated increased risk of sepsis associated with elevated C-reactive protein, temperature, white blood cell count, creatinine, lactate level, SOFA score, and detailed medical history score. In contrast, normal or high blood pressure values (systolic/diastolic) had strong negative coefficients, suggesting a protective effect (see Table 15). Arterial blood gas and platelet count variables also lowered the predicted likelihood of sepsis when values were within normal range. Chills severity appeared negatively weighted, likely due to its frequent encoding or lower overall predictive contribution.

The decision tree model placed overwhelming reliance on systolic blood pressure to initiate predictive splits, followed by minor contributions from glucose level and fever severity. Palpitations and chills appeared, but their influence was minimal, underscoring the tree’s sensitivity to structured vital signs rather than subjective symptom data.

Table 16 highlights the most influential features in the decision tree model trained on the combined dataset, with systolic blood pressure emerging as the dominant split criterion, followed by glucose level and fever severity with progressively lower impact.

Overall, the integration of data from urgent care, ambulance, and hospital emergency department stages significantly improves model performance and interpretability. Key features span vital signs, laboratory results, symptom scores, and organ function indices, forming a multidimensional view of patient status. White-box models not only provide clinical transparency but also demonstrate strong predictive utility in identifying sepsis risk throughout early and acute care transitions.

### 4.2. Black Box Analysis

Black-box analysis in ML, particularly using feed-forward neural networks, offers powerful predictive capabilities in emergency medicine when applied across urgent care, ambulance, and hospital emergency department datasets. These models can learn complex, nonlinear relationships between physiological signals, clinical observations, and outcomes such as deterioration risk or hospital emergency department admission, but their internal decision-making processes remain opaque to clinicians. By ingesting data from wearable devices at urgent care, real-time vitals during ambulance transport, and diagnostic inputs upon hospital arrival, feed-forward networks can generate rapid predictions that support triage and escalation decisions. However, their lack of interpretability poses challenges for clinical trust and accountability, especially in high-stakes environments. Despite this, their ability to integrate multimodal data and uncover subtle patterns makes them valuable tools for early warning systems and outcome forecasting, provided that safeguards like post-hoc explainability methods or hybrid modeling approaches are in place.

#### 4.2.1. Urgent Care

A feedforward neural network built using the Keras Sequential API was deployed to explore the predictive utility of the urgent care assessment dataset for early sepsis detection. This dataset contains structured symptom severity scores ranging from 0 to 3, capturing signs such as fever, chills, breathing difficulty, and cognitive changes, all self-reported prior to formal medical contact.

To prepare the data, all input features were standardized using *StandardScaler*, improving training efficiency and stability. The network architecture consisted of two hidden layers with 64 and 32 neurons, respectively, combined with dropout rates of 0.3 and 0.2 to reduce overfitting. The model was trained using the Adam optimizer (learning rate = 0.001) and binary crossentropy as the loss function. Early stopping was applied based on validation loss to avoid unnecessary epochs.

Holdout evaluation demonstrated promising results, with an accuracy of 0.82 and an AUC of 0.91, as summarized in Table 17. These figures suggest that the symptom severity scores contain latent nonlinear patterns indicative of sepsis risk in a static setting.

However, performance dropped significantly during stratified five-fold cross-validation, revealing an average accuracy of 47.1% and mean AUC of 49.81%. This sharp contrast suggests poor generalization and high model variance across splits, likely attributable to the subjective and limited scope of self-reported symptom data.

Despite these limitations, the model achieved relatively high precision (0.85) for identifying non-sepsis cases in the holdout set. This indicates possible utility in low-risk patient triage, potentially reducing the burden of false positives and streamlining care escalation in remote settings.

#### 4.2.2. Urgent Care and Ambulance Analysis

A feedforward neural network was applied to jointly model symptom severity scores from the urgent care assessment dataset and physiological measurements from the Ambulance stage, thereby capturing both subjective and objective indicators relevant to early sepsis detection. This hybrid dataset enabled the network to learn from a broader spectrum of clinical signals, ranging from patient-reported symptoms to vital signs collected en route to the hospital emergency department.

All input features were standardized using *StandardScaler* to enhance training efficiency. The model architecture incorporated two hidden layers with 64 and 32 neurons, each utilizing ReLU activations. Dropout layers with a rate of 0.5 were included after each hidden layer to mitigate overfitting, and the output node used sigmoid activation for binary classification. Optimization was performed using the Adam optimizer and binary crossentropy loss over 50 epochs, with stratified train-test splitting to maintain class balance.

The model achieved exceptional performance, with an overall accuracy of 99% and AUC of 98%, signaling high discriminative power in identifying sepsis cases using the combined data. Table 18 summarizes these results.

The minimal performance drop in one fold suggests strong generalization capability, especially when contrasted with the standalone urgent care assessment model, which showed high variance and poor generalization in cross-validation. This dramatic improvement highlights the value of combining early-stage symptom scoring with real-time ambulance physiological inputs. Notably, dropout regularization proved effective in preventing overfitting despite the relatively small data size.

Clinically, this model enables actionable sepsis risk stratification before hospital arrival, integrating patient self-report with emergent clinical observations. Such stage-aware modeling is critical for triage decisions, prioritizing interventions en route, and improving outcomes in pre-hospital care. The fusion of subjective and objective signals reflects the strength of multi-modal neural architectures in predictive emergency medicine.

#### 4.2.3. Urgent Care, Ambulance and Hospital Emergency Department Analysis

A feedforward neural network was trained using a comprehensive dataset that integrates structured features from the urgent care, ambulance, and hospital emergency department assessment stages. These data span the full trajectory of early patient monitoring, from subjective symptom reporting in the urgent care setting to vital signs and clinical observations captured during ambulance transport and culminating in laboratory findings and scoring systems recorded at hospital emergency department admission. The unified dataset allowed the model to learn across a continuum of care, using both self-reported and objective physiological indicators relevant to sepsis progression.

All input variables were standardized using *StandardScaler* to ensure consistent feature scaling across modalities. The architecture comprised two hidden layers with 64 and 32 neurons, each activated by ReLU and followed by dropout layers set at 0.5 to prevent overfitting. The final output layer used sigmoid activation for binary classification. Training was conducted over 50 epochs using the Adam optimizer and binary crossentropy as the loss function. Stratified train-test splitting maintained balance across the sepsis-positive and negative classes.

Model performance was strong and consistent, with overall accuracy reaching 99% and AUC at 98%, as shown in Table 19. Evaluation across five folds demonstrated perfect prediction in four folds, with one fold showing minor performance degradation due to a single misclassification.

These results demonstrate the model’s strong generalization capability and confirm that robust decision boundaries were learned by combining diverse feature types. The inclusion of high-resolution hospital data, such as lactate levels, C-reactive protein, SOFA scores, and arterial blood gas measurements, enhanced the model’s ability to capture systemic dysfunction patterns indicative of sepsis. When paired with earlier-stage vitals and symptoms, these features contributed to a multi-tiered representation of risk.

Clinically, this end-to-end modeling approach provides substantial value for emergency triage and early intervention planning. It supports continuous sepsis risk assessment from the moment symptoms are reported at urgent care, through ambulance transit, and into hospital emergency department diagnosis, delivering high-confidence alerts at critical decision points. The high classification accuracy across all validation folds reinforces the feasibility of deploying neural architectures within real-world emergency care systems, particularly for dynamic prediction during patient transport and admission.

## 5. Conclusions and Future Work

Predictive modeling for early sepsis detection was evaluated using both interpretable white-box models and a black-box neural network, across three stages of emergency care: urgent care, ambulance, and hospital emergency department. Each stage introduced unique data modalities ranging from self-reported symptoms to objective clinical and laboratory features.

At the urgent care stage, where input features were limited to subjective symptom severity scores, models showed modest performance. White-box algorithms like decision tree and random forest achieved 82% accuracy and 91% AUC, while the neural network matched these metrics in holdout evaluation but struggled with generalization during cross-validation. This suggests limited reliability when relying solely on self-reported data.

Incorporating ambulance data notably enhanced prediction. The addition of vital signs and transit-time assessments led white-box models to reach nearly perfect performance, up to 99% accuracy and 99% AUC. The black-box neural network mirrored these outcomes, capturing more complex interactions and demonstrating better generalization. Objective features proved critical in reducing variance and improving consistency.

When hospital emergency department-level data was added, both model types achieved their highest accuracy and AUC scores. White-box models remained highly interpretable while maintaining metrics above 98%. The neural network slightly outperformed its counterparts with an accuracy of 99.32% and AUC of 98.62%, showing strong generalization across validation folds. The integration of biochemical markers (i.e., CRP, lactate, ABG values) reinforced the value of deep multimodal feature learning.

Overall, white-box models provide transparency and clinical trust, especially valuable for decision-making. However, black-box architectures offer enhanced predictive capability when combining heterogeneous data across the full care pathway, supporting scalable and high-confidence deployment in real-world triage systems.

Future work will focus on clinical translation pathways, including prospective trials, integration with electronic health records, deployment in ambulance monitoring systems, and evaluation of ethical and regulatory considerations to support real-world implementation, while also exploring direct comparisons between model predictions and clinical judgments made by human experts at each stage of care. Evaluating physicians’ diagnostic performance based on urgent care-reported symptoms, ambulance assessments, and comprehensive hospital emergency department data will provide deeper insight into how AI mechanisms align with or exceed human intuition and clinical experience, ultimately informing hybrid decision-support systems that enhance safety, efficiency, and trust in high-stakes medical environments. Additionally, as part of future work, we plan to conduct statistical comparisons between white-box and black-box models, such as paired tests on AUC, to determine the significance of observed performance differences and further justify the use of more complex modeling approaches. Similarly, the discussion and analysis of multiple technical details including training and validation conversion are also focus of future work.

To strengthen clinical integration, each model could be tailored to its respective care environment. The urgent care model, built on symptom severity scores, lends itself well to a patient facing mobile application or kiosk interface, allowing individuals to self report symptoms and receive preliminary risk assessments before clinical evaluation. The ambulance stage model, enriched with vital signs and transit time data, could be deployed on rugged tablets used by paramedics, offering real time triage support during transport and enabling early alerts to receiving hospitals. At the hospital emergency department level, the model could be embedded within the *Electronic Health Record* (EHR) system, functioning as a dynamic alert mechanism that flags high risk patients based on incoming lab results and clinical observations. This tiered deployment strategy ensures that predictive insights are delivered at the point of care, enhancing decision making across the full emergency response continuum.

## Figures and Tables

**Table 1:** Comparison of ML techniques for sepsis detection.

Technique	Study	Accuracy	AUC	Precision	Recall
Random Forest (RF)	[4]	99%	99.99%	–	–
RF + SMOTE	[24]	95%	98%	98%	91%
XGBoost	[21]	–	79%	–	–
XGBoost + Temporal Features	[21]	–	92% (Septic Shock)	–	–
Balanced Bagging	[6]	96%	–	–	–
SVM (Thermal Imaging)	[9]	80%	–	–	–
HHO + SVM	[23]	97%	96%	–	–
Gradient Boosting	[5]	92%	91%	86%	89%
ANN (MIMIC-III)	[26]	90%	82%	–	90%
CNN (PhysioNet)	[18]	99.99%	–	–	–
LSTM (PhysioNet)	[18]	99.96%	–	–	–
MLP (PhysioNet)	[27]	94%	–	–	–
MLP (MIMIC-III)	[27]	83%	–	–	–
RF (Pediatrics)	[22]	92%	86%	–	–
RF + SHAP (SAE)	[25]	92%	84%	–	–

**Table 2:** Feature composition of the urgent care assessment dataset.

Category	Features
Symptom Severity Scores (0–3)	Fever Severity, Altered Mental Status Severity, Palpitations Severity, Chills Severity, Breathing Difficulty Severity, Skin Discoloration Severity, Fatigue Severity, Confusion Severity
Outcome Variable	SEPSIS (binary: Yes/No)

**Table 3:** Representative urgent care assessment record.

Symptom	Severity Score
Fever	2
Altered Mental Status	1
Palpitations	0
Chills	2
Breathing Difficulty	1
Skin Discoloration	0
Fatigue	2
Confusion	0
Sepsis Diagnosis	Yes

**Table 4:** Feature composition of the ambulance assessment dataset.

Category	Features
Symptom Severity Scores (0–3)	Fever Severity, Altered Mental Status Severity, Palpitations Severity, Chills Severity, Breathing Difficulty Severity, Skin Discoloration Severity
Vital Signs and Lab Indicators	Systolic Blood Pressure, Diastolic Blood Pressure, Heart Rate, Respiratory Rate, Oxygen Saturation, Temperature
Clinical Observations	Glucose Level, Capillary Refill Time, Glasgow Coma Scale
Outcome Variable	SEPSIS (binary: Yes/No)

**Table 5:** Representative ambulance assessment record.

Measurement	Value
Fever Severity	3
Altered Mental Status	1
Palpitations	1
Chills	1
Breathing Difficulty	1
Skin Abnormality	1
Systolic Blood Pressure	83 mmHg
Diastolic Blood Pressure	59 mmHg
Heart Rate	131 bpm
Respiratory Rate	33.5 breaths/min
Oxygen Saturation	94.26%
Glucose Level	138.87 mg/dL
Capillary Refill Time	3.35 s
Glasgow Coma Scale	14.51
Temperature	38.90 ∘C
SEPSIS Diagnosis	Yes

**Table 6:** Feature composition of the hospital emergency department assessment dataset.

Category	Features
Symptom Severity Scores (0–3)	Fever Severity, Altered Mental Status Severity, Palpitations Severity, Chills Severity, Breathing Difficulty Severity, Skin Discoloration Severity, Fatigue Severity, Confusion Severity
Clinical Assessment Scores (0–3)	Auscultation Score, Detailed Medical History Score, SOFA Score
Laboratory Biomarkers	White Blood Cell Count, Lactate Level, C-Reactive Protein, Procalcitonin, Creatinine, Bilirubin, Platelet Count
Blood Gas Analysis	Arterial Blood Gas (pH), Arterial Blood Gas (pCO_2_), Arterial Blood Gas (pO_2_)
Microbiological Results	Blood Culture Results (Negative/Positive)
Outcome Variable	SEPSIS (binary: Yes/No)

**Table 7:** Representative hospital emergency department assessment record.

Measurement	Value
Fever Severity	3
Altered Mental Status	2
Palpitations	2
Chills	2
Breathing Difficulty	2
Fatigue	3
Confusion	2
Auscultation Score	2
Detailed Medical History Score	3
White Blood Cell Count	20.026 × 10^3^/μL
Lactate Level	4.024 mmol/L
C-Reactive Protein	115.001 mg/L
Procalcitonin	3.437 ng/mL
Creatinine	2.147 mg/dL
Bilirubin	2.081 mg/dL
Platelet Count	121.39 × 10^3^/μL
SOFA Score	4.802
Blood Culture Results	Negative
Arterial Blood Gas (pH)	7.254
Arterial Blood Gas (pCO_2_)	42.161 mmHg
Arterial Blood Gas (pO_2_)	55.201 mmHg
SEPSIS Diagnosis	Yes

**Table 8:** Model performance on urgent care assessment dataset.

Model	Mean Accuracy	Mean AUC
Logistic Regression	82%	90%
Decision Tree	75%	83%
Random Forest	82%	90%

**Table 9:** Top Features by model (urgent care assessment).

Decision Tree	Random Forest	Logistic Regression
Fever Severity (0.59)	Fever Severity (0.39)	Chills Severity (1.26)
Breathing Difficulty Severity (0.19)	Chills Severity (0.24)	Skin Discoloration Severity (0.60)
Chills Severity (0.18)	Breathing Difficulty Severity (0.21)	Fever Severity (0.57)
Skin Discoloration Severity (0.04)	Fatigue Severity (0.07)	Altered Mental Status Severity (−0.47)
Altered Mental Status Severity (0.00)	Skin Discoloration Severity (0.06)	Breathing Difficulty Severity (0.30)
Palpitations Severity (0.00)	Altered Mental Status Severity (0.02)	Fatigue Severity (0.15)
Fatigue Severity (0.00)	Palpitations Severity (0.00)	Palpitations Severity (0.00)
Confusion Severity (0.00)	Confusion Severity (0.00)	Confusion Severity (0.00)

**Table 10:** White-box model performance on urgent care + ambulance dataset.

Model	Mean Accuracy	Mean AUC
Logistic Regression	99%	98%
Decision Tree	98%	98%
Random Forest	99%	99%

**Table 11:** Top predictive features from random forest (urgent care + ambulance).

Feature	Importance Score
Capillary Refill Time	Highest
Temperature	High
Systolic Blood Pressure	High
Diastolic Blood Pressure	High
Oxygen Saturation	Moderate
Heart Rate	Moderate
Respiratory Rate	Moderate
Glucose Level	Moderate
Glasgow Coma Scale	Low
Chills Severity	Low

**Table 12:** Feature coefficient summary—logistic regression.

Feature	Coefficient Direction
Temperature	Strong Positive
Capillary Refill Time	Positive
Heart Rate	Positive
Systolic Blood Pressure	Strong Negative
Diastolic Blood Pressure	Strong Negative
Chills Severity	Mild Positive
Oxygen Saturation	Mild Positive
Breathing Difficulty Severity	Mild Positive
Fever Severity	Mild Positive

**Table 13:** White-box model performance on urgent care + ambulance + hospital emergency department dataset.

Model	Mean Accuracy	Mean AUC
Logistic Regression	99%	98%
Decision Tree	98%	98%
Random Forest	99%	99%

**Table 14:** Top predictive features—random forest (combined dataset).

Feature	Clinical Relevance
Temperature	Core infection marker (fever)
Heart Rate	Cardiovascular stress
Lactate Level	Tissue hypoperfusion/metabolic acidosis
Diastolic Blood Pressure	Hemodynamic instability
C-Reactive Protein	Systemic inflammation
Arterial Blood Gas (pO_2_)	Oxygenation status
White Blood Cell Count	Immune activation
Platelet Count	Coagulation & immune response
SOFA Score	Organ dysfunction index
Oxygen Saturation	Blood oxygen delivery

**Table 15:** Top logistic regression coefficients (combined dataset).

Feature	Coefficient Direction
C-Reactive Protein	Strong Positive
Temperature	Positive
White Blood Cell Count	Positive
Creatinine	Positive
SOFA Score	Positive
Lactate Level	Positive
Detailed Medical History Score	Positive
Systolic Blood Pressure	Strong Negative
Diastolic Blood Pressure	Strong Negative
Platelet Count	Negative
Chills Severity	Negative

**Table 16:** Top Decision Tree Splits (Combined Dataset).

Feature	Relative Impact
Systolic Blood Pressure	Dominant
Glucose Level	Minor
Fever Severity	Minimal
Palpitations Severity	Negligible
Chills Severity	Negligible

**Table 17:** Neural network performance—urgent care assessment.

Evaluation Method	Accuracy	AUC
Holdout Test Set	82%	91%

**Table 18:** Neural network performance—combined urgent care and ambulance data.

Evaluation Method	Accuracy	AUC
Holdout Test Set	99%	98%

**Table 19:** Neural network performance—combined urgent care, ambulance, and hospital emergency department data.

Evaluation Method	Accuracy	AUC
Holdout Test Set	99%	98%

## Data Availability

The datasets generated during the current study are available from the corresponding author on reasonable request.
